# Hybridization facilitates evolutionary rescue

**DOI:** 10.1111/eva.12214

**Published:** 2014-09-25

**Authors:** Rike B Stelkens, Michael A Brockhurst, Gregory D D Hurst, Duncan Greig

**Affiliations:** 1 Institute of Integrative Biology, University of LiverpoolLiverpool, UK; 2 Department of Biology, University of YorkYork, UK; 3 Max Planck Institute for Evolutionary BiologyPlön, Germany; 4 The Galton Laboratory, Department of Genetics, Evolution, and Environment, University College LondonLondon, UK

**Keywords:** evolutionary rescue, extinction, genetic variation, global change, hybridization, *Saccharomyces*

## Abstract

The resilience of populations to rapid environmental degradation is a major concern for biodiversity conservation. When environments deteriorate to lethal levels, species must evolve to adapt to the new conditions to avoid extinction. Here, we test the hypothesis that evolutionary rescue may be enabled by hybridization, because hybridization increases genetic variability. Using experimental evolution, we show that interspecific hybrid populations of *Saccharomyces* yeast adapt to grow in more highly degraded environments than intraspecific and parental crosses, resulting in survival rates far exceeding those of their ancestors. We conclude that hybridization can increase evolutionary responsiveness and that taxa able to exchange genes with distant relatives may better survive rapid environmental change.

## Introduction

Current rates of global change are likely to exceed the rate at which species can evolve (Parmesan 1996; Lindsey et al. 2013). If plastic responses and dispersal are also limited, population extinction will occur (Pounds et al. 2006; Sinervo et al. 2010). In accordance with theory (Gomulkiewicz and Holt 1995; Orr and Unckless 2008; Chevin 2013), experiments have shown that evolutionary rescue depends on the rate and severity of environmental degradation (Lindsey et al. 2013; Uecker et al. 2014), population size (Bell and Gonzalez 2009; Ramsayer et al. 2013) and migration rates (Bell and Gonzalez 2011). Standing genetic variation could also help evolutionary rescue by speeding adaptation (Burger and Lynch 1995; Lande and Shannon 1996; Orr and Unckless 2008), because alleles that are beneficial in the new environment are available immediately and at higher frequencies than can be provided by *de novo* mutation (Barrett and Schluter 2008; Hedrick 2013). Hybrid populations that have previously undergone admixture with distant populations or with sister species contain large amounts of genetic variation (Dettman et al. 2008), and there is increasing evidence that hybridization helps adaptation and speciation (Rieseberg et al. 2003; Seehausen 2004; Arnold 2006; Abbott et al. 2013).

We use experimental evolution in increasingly harmful environments to test if interspecific F2 hybrid populations of *Saccharomyces* yeast are able to adapt to more extreme conditions than either intraspecific F2 crosses or F1 hybrids and parental genotypes. Experimental evolution with *Saccharomyces* yeast is ideal for comparing the evolutionary performance of hybrids versus nonhybrids in changing environments. Diploid cells can reproduce rapidly by asexual mitosis, dividing as frequently as every 2 h, but they can also be induced to enter meiosis and produce haploid sexual gametes which can fuse with each other to make new diploids (self-fertilize) or with gametes from other strains or species, making F1 diploids (Fig. S1). We made a set of F1 diploids whose parents differed by between 0.06% (*S. paradoxus* × *S. paradoxus* intraspecific crosses) and 14% (*S. paradoxus* × *S. cerevisiae* interspecific crosses) genome-wide sequence divergence (Table 1), and induced meiosis and haploid fusion to generate F2 populations whose members varied according to the genetic differences they inherited from their diverged parents. Hybrid and nonhybrid F2, F1 and parental populations were grown and transferred into growth media supplemented with increasing amounts of salt to simulate a habitat that gradually deteriorates in quality. We found that hybrid survival rates exceeded those of their ancestors by far. We conclude that hybridization can increase evolutionary responsiveness to environmental change and that taxa able to exchange genes with distant relatives may better survive rapid environmental change.

**Table 1:** List of intraspecific and interspecific crosses

Cross no.	Parent 1	sp.	NCYC accession	Parent 2	sp.	NCYC accession	Genetic distance	Phenotypic distance
1	YPS138	*par*	3711	DBVPG6304	*par*	3685	0.0021	3.78
2	YPS138	*par*	3711	273614N	*cer*	3585	0.1384	2.96
3	Y7	*par*	3664	Y8.1	*par*	3707	0.0008	1.17
4	Y7	*par*	3691	BC187	*cer*	3591	0.1358	3.93
5	CBS432	*par*	3662	IFO1804	*par*	3715	0.0119	3.85
6	CBS432	*par*	3662	YJM978	*cer*	3617	0.1369	4.17
7	CBS5829	*par*	3682	N-17	*par*	3708	0.0013	1.34
8	CBS5829	*par*	3682	UWOPS05227.2	*cer*	3629	0.1366	3.09
9	Y9.6	*par*	3673	N-17	*par*	3708	0.0014	3.04
10	Y9.6	*par*	3673	UWOPS05227.2	*cer*	3629	0.1359	3.54
11	DBVPG6304	*par*	3685	Y6.5	*par*	3697	0.0373	4.04
12	DBVPG6304	*par*	3712	NCYC110	*cer*	3601	0.1405	5.66
13	Q32.3	*par*	3665	YPS138	*par*	3711	0.0366	2.70
14	Q32.3	*par*	3665	273614N	*cer*	3611	0.1335	2.99
15	Q74.4	*par*	3674	N-44	*par*	3714	0.0117	1.44
16	Q74.4	*par*	3674	DBVPG1106	*cer*	3621	0.1334	3.07
17	KPN3829	*par*	3710	N-17	*par*	3681	0.0012	3.45
18	KPN3829	*par*	3683	NCYC110	*cer*	3626	0.1363	4.11
19	N-44	*par*	3687	CBS432	*par*	3689	0.0122	2.84
20	N-44	*par*	3714	YPS128	*cer*	3607	0.1357	2.47
21	IFO1804	*par*	3715	CBS5829	*par*	3682	0.0118	2.44
22	IFO1804	*par*	3715	YIIc17_E5	*cer*	3586	0.1347	7.22
23	Y9.6	*par*	3700	Z1.1	*par*	3669	0.0010	1.93
24	Y9.6	*par*	3673	YJM978	*cer*	3617	0.1353	3.00
25	YPS138	*par*	3711	Y9.6	*par*	3673	0.0380	3.02
26	YPS138	*par*	3711	L-1374	*cer*	3598	0.1378	3.27
27	YPS138	*par*	3711	KPN3829	*par*	3683	0.0372	3.59
28	YPS138	*par*	3711	NCYC110	*cer*	3601	0.1398	4.97
29	CBS5829	*par*	3682	Y8.1	*par*	3707	0.0012	1.18
30	CBS5829	*par*	3682	DBVPG6044	*cer*	3625	0.1375	2.38
31	Y9.6	*par*	3673	DBVPG6304	*par*	3712	0.0377	4.48
32	Y9.6	*par*	3700	DBVPG1373	*cer*	3595	0.1342	2.91

*par* = *S. paradoxus*; *cer* = *S. cerevisiae*.

## Materials and methods

### Parental strains

We used 26 parental strains of *S. cerevisiae* or *S. paradoxus* from the National Collection of Yeast Cultures (NCYC; http://www.ncyc.co.uk/; Table 1). The 26 perfectly homozygous (except at the mating type locus) homothallic diploid strains were previously derived by monosporic cloning of isolates (Fig. S1, parts 1–3) originally collected from ecologically widely diverse habitats across the world, so that the whole set contains a wide range of variation (Liti et al. 2009). We previously estimated phenotypic distances (PD, Table 1) from the multivariate growth data collected in seven different environments (Stelkens et al. 2014a). Our PD matched the distances calculated from previously published multivariate phenotypes (*R*^2^ = 0.42, F_1,45_ = 30.87, *P* < 0.001), which were calculated using over 600 different environments (Warringer et al. 2011).

### F1 strains

From the 26 homozygous parental strains, we selected 16 *S. paradoxus* reference parents (‘parent 1’, Table 1) and paired each with both a different *S. paradoxus* parent and an *S. cerevisiae* parent (‘parent 2’, Table 1, Fig. S1) to make 32 pairs, 16 intraspecific and 16 interspecific. Isogenic heterothallic and genetically marked haploid strains were previously produced from each of the diploid parents by deleting their *HO* and *URA3* loci with the drug resistance markers *HygMX* and *KanMX,* respectively (Cubillos et al. 2009; Liti et al. 2009). We used these haploid derivatives of the parent strains to make pure clones of diploid F1 heterozygotes from each pair (Fig. S1, part 4). Parental strains were paired as shown in Table 1. The haploids were grown from frozen samples and incubated at 30**°**C in 10 mL YEPD (1% yeast extract, 2% peptone, 2% dextrose) in a shaking incubator for 24 h. Diploid F1 hybrids were made by mixing equal volumes of two haploid parental strains of different mating types and incubating the mixture on YEPD plates (with the addition of 2.5% agar) overnight. The mixed culture of F1 diploids and unmated parental haploids was streaked to new YEPD plates and grown for 48 h. The resulting colonies, each derived from a single cell, were replica-plated to KAC agar plates (2% potassium acetate, 2% agar) and incubated for 48 h at 25**°**C to induce sporulation. Sporulating colonies were microscopically identified as F1 hybrids (diploids can sporulate, but haploids cannot). A pure F1 diploid colony from each pair of diploid parents was picked from the YEPD plate, propagated clonally in YEPD and frozen for later use.

### F2 populations

Each F1 hybrid strain was spread on to a new YEPD plate, grown for 48 h, replica-plated to KAC and incubated at 25**°**C for 5 days to obtain a large sample of F1 haploid spores (Fig. S1. part 5). To remove any remaining F1 diploid cells that had not undergone meiosis, cells and spores were scraped off the KAC plates, suspended in 1 mL H_2_O, spun down, resuspended in 1 mL 0.1825N NaOH and shaken in a heat block at 1000 rpm for 10 min at 30**°**C. To neutralize, 1 mL 0.1825N HCl was immediately added, spun down, and resuspended in 1 mL H_2_O. These F1 haploid spores were then frozen and used in the experiment to found diploid F2 populations (Fig. S1, parts 6 & 7).

### Serial transfer in deteriorating environment

We grew the populations in growth medium supplemented with gradually increasing amounts of NaCl to simulate a habitat that gradually deteriorates in quality. Previous research has shown that the osmotic and ionic stress caused by salt inhibits growth in yeast (Hohmann 2002) and that the ability of yeast to cope with salt stress is a quantitative trait, likely influenced by many genes with small effect (>500 genes; Warringer et al. 2003). In our experiment, concentrations larger than 10 g/L reduced the growth of parental strains, and the final concentration of 160 g/L was lethal and caused complete extinction, confirming findings of another study using *S. cerevisiae* (Bell and Gonzalez 2009).

The wells of 96-well flat-bottomed culture plates were filled with 180 μL growth medium (MIN + URA, 0.67% yeast nitrogen base without amino acids, 2% glucose, 2% agar, 0.003% uracil). The addition of uracil was necessary because all nonparental strains in this experiment were uracil auxotrophs (*ura3::KanMX*). The central 60 wells of the culture plates were then inoculated with 20 μL yeast culture containing F1 spores (Fig. S2). Because sporulation efficiency of F1 hybrids and spore viability varied between crosses, we standardized population size across wells before the start of the experiment. Samples of F1 spores from each cross were plated out on YEPD agar and colony counts were used to dilute the inoculation suspension so that approximately 10 viable F2 hybrids were used per well, representing a founder population.

Of the 60 populations on each plate, half of the wells (*n* = 30) contained spores from a single intraspecific cross and half contained spores from the corresponding interspecific cross (Table 1). From here on, we refer to the 30 replicate F2 populations of the same cross as ‘meta-population’. To cancel out positional effects, the two F2 crosses were distributed symmetrically on the plate (e.g. interspecific populations on the left, intraspecific strains on the right half). In total, we tested 960 F2 populations (32 different hybrid strains in 16 pairwise intra- versus interspecific combinations).

Wells on the first culture plate (P1) contained MIN + URA. Populations were given 72 h on P1, providing enough time for spore germination to form the F2 generation, and growth to stationary phase. Then, they were transferred to a new culture plate (P2), containing MIN + URA and salt (40 g/L NaCl; see serial transfer scheme in Fig. S2). At each transfer, populations were diluted 100-fold to see whether they would recover again from rare. After 72 h on P2, populations were transferred to a new plate (P3) containing a higher salt concentration (80 g/L). After 72 h on P3, populations were transferred to a new plate (P4) containing the same medium with the same concentration of salt (80 g/L). This transfer into identical environmental conditions allowed for a ‘recovery phase’, that is it allowed those individuals with beneficial alleles to increase in the population. After 72 h on P4, populations were transferred to a new plate (P5) with a higher salt concentration (120 g/L). After 72 h on P5, we again allowed for two rounds of ‘recovery’ in identical salt conditions on plates P6 and P7 (120 g/L), to allow for fixation of beneficial alleles. After 72 h on each P5, P6 and P7, populations were transferred to a new plate (P8) with higher salt concentration (160 g/L). After 72 h and transfer to a new plate (P9) with the same salt concentration (160 g/L), all populations were extinct and the experiment was stopped.

For comparison, the same experimental procedure was applied to the 32 F1 hybrid crosses and the 26 parental strains. Instead of using 30 populations per cross as in F2 hybrids, we used only six populations per strain (totalling 192 F1 and 156 parental populations) because F1 hybrid and parental genotypes are genetically uniform clones (except for any new random mutations), hence the populations' variance in response to stress was expected to be low and sufficiently captured in fewer replicates.

### Measuring survival

At the beginning (0 h) and at the end (72 h) of the growth period on each plate, optical density (OD_600_) in every well was measured with a microplate reader (Infinite M200 Pro, Tecan, Reading, UK). Populations (i.e. wells) were considered extinct when there was no increase in OD between 0 and 72 h, after correcting for background noise (using the highest OD measured in 36 control wells containing the growth medium but no yeast). The overall survival rate of a meta-population of a given cross was calculated as the number of populations that survived/total number of replicate populations (*n* = 30).

### Statistical analysis

Hypothesis testing was performed using R. We used a series of generalized linear mixed effect models (GLMMs, lme package; Bates and Maechler 2009) to test for variance in *survival* (with binomial fit) between intra- and interspecific crosses, in the F1 and F2 generation separately. The base model contained cross *type* (intra- or interspecific), number of *days* in experiment (7 levels) and their interaction (days*type) as fixed effects and *population* (*n* = 1308), *parent 1* (the ‘reference’ parent) and *parent 2* as random effects. Another series of GLMMs was used to test for variance in *survival* between generations, using *generation* (parental, F1 or F2 hybrid), and *days* in experiment (7 levels) as fixed effects and *population*, *parent 1* and *parent 2* as random effects.

To evaluate the explanatory importance of each variable, alternative models with or without this variable were compared, using log-likelihood ratio tests (LRT) with restricted maximum likelihood (REML; Zuur et al. 2009). If an alternative model had a significantly better fit, this model was subsequently compared against further reduced models.

## Results

**Figure 1:**
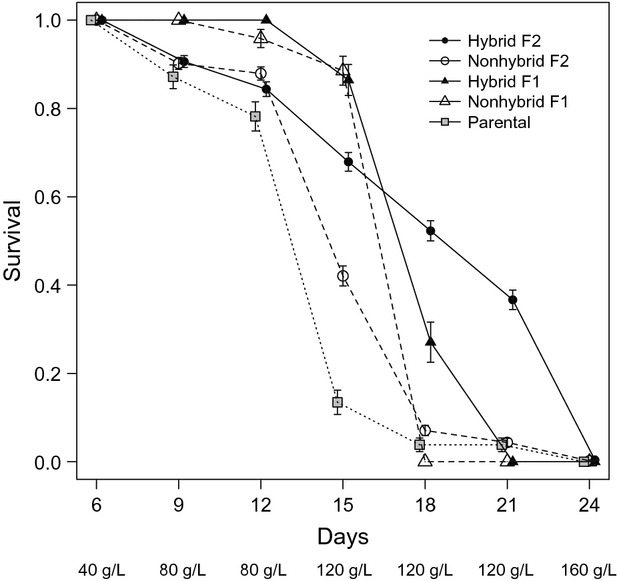
Mean survival of hybrids and nonhybrids in deteriorating environment. Solid lines with filled symbols represent hybrid populations (those with parents from different species), dashed lines and open symbols represent nonhybrid populations (with parents of the same species), and the dotted line with grey squares represents parental populations. Triangles are the F1 populations; circles are the F2 populations. Error bars are standard errors. The amount of salt in the growth medium is shown below the *x*-axis.

As salt concentration increased through time, the survival of all types of populations decreased significantly (Fig. 1, factor *days* in Tables 2 and 3), an expected effect of lethal osmotic stress (Bell and Gonzalez 2009, 2011). The resilience of F2 hybrid populations was greater than that of nonhybrids (Fig. 1), with a significantly larger proportion of F2 hybrid populations surviving the high salt environment than F2 nonhybrid populations (factor *cross type,* Table 2). This is consistent with our prediction that hybrid crosses with genetically distant parents produce new genetic combinations with more pre-adaptations for survival under stressful conditions than the nonhybrid crosses with genetically closer parents, increasing their likelihood for evolutionary rescue. The significant interaction between increasing salt and cross type (days*type) confirms the difference in response of hybrids and nonhybrids to environmental stress. We also found significant differences in survival between replicate F2 populations (factor *population,* Table 2), consistent with founder effects from sampling a small number (10) of highly variable F2 genotypes in each experimental population.

**Table 2:** Comparison between hybrids and nonhybrids

Effect tested	Fixed effects	Random effects	AIC	χ^2^	d.f.	*P*
F2						
	D, T, D^*^T	**P, P1, P2**	**2759.9**			
*days* (D)	T	P, P1, P2	8645.7	5875.7	12	<0.001
*cross type* (T)	D	P, P1, P2	2940.3	194.4	7	<0.001
days^*^type (D^*^T)	D, T	P, P1, P2	2924.2	176.4	6	<0.001
*population* (P)	D, T, D^*^T	P1, P2	3006.1	248.2	1	<0.001
*parent 1* (P1)	D, T, D^*^T	P, P2	3027.9	270	1	<0.001
*parent 2* (P2)	D, T, D^*^T	P, P1	2787.8	30.0	1	<0.001
F1						
	D, T, D^*^T	**P, P1, P2**	**234.2**			
*days* (D)	T	P, P1, P2	285.9	1634.2	12	<0.001
*cross type* (T)	**D**	**P, P1, P2**	**233.8**	13.7	7	0.06
days^*^type (D^*^T)	**D, T**	**P, P1, P2**	**232.8**	10.6	6	0.10
*population* (P)	**D**	**P1, P2**	**231.8**	0.0	1	0.99
*parent 1* (P1)	D	P2	234.9	5.1	1	0.02
*parent 2* (P2)	D	P1	317.1	87.3	1	<0.001

Likelihood ratio tests comparing generalized linear mixed models (GLMMs) on the effects of the number of *days* in the experiment, cross *type* (inter- or intraspecific), their interaction (days^*^type), *population* (*n* = 1308), *parental strain 1* (the same in both inter-and intraspecific crosses) and *parental strain 2* on survival in deteriorating environments. The upper part of the table shows the analysis within F2 hybrids; the lower part shows F1 hybrids. Akaikes information criterion (AIC) describes the quality of fit of each model. To evaluate the significance of fixed and random effects, alternative models without the variable of interest were compared to the full model (bold) using likelihood ratio tests. If an alternative model had a significantly better fit (bold), this model was subsequently compared against further reduced models.

**Table 3:** Comparison between generations

Effect tested	Fixed effects	Random effects	AIC	χ^2^	d.f.	*P*
	**D, G**	**P, P1, P2**	**3881.9**			
*days* (D)	G	P, P1, P2	11986.6	8116.7	6	<0.001
*generation* (G)	D	P, P1, P2	3891.6	13.7	2	0.001
*population* (P)	D, G	P1, P2	4168.2	288.3	1	<0.001
*parent 1* (P1)	D, G	P, P2	4421.1	541.1	1	<0.001
*parent 2* (P2)	D, G	P, P1	4022.9	143	1	<0.001

Likelihood ratio tests comparing generalized linear mixed models (GLMMs) on the effects of the number of *days* in experiment, *generation* (parental, F1 or F2), *population* (*n* = 1308), *parental strain 1* (the same in both inter-and intraspecific crosses) and *parental strain 2* on survival in deteriorating environments. Effect evaluation as in Table 1. To evaluate the significance of fixed and random effects, alternative models without the variable of interest were compared to the full model (bold) using likelihood ratio tests. If an alternative model had a significantly better fit (bold), this model was subsequently compared against further reduced models.

In the F1 generation, no differences were found in the survival of hybrids and nonhybrids, and the interaction effect was not significant. This suggests that the benefit that hybrids gain in the F2 generation comes not only from great genetic differences between their parents (differences which are also present in the F1), but also from the loss of some alleles and homozygosis of other alleles by random recombination, segregation and syngamy. Concordantly, the *population* effect was absent in the F1, which is consistent with a lack of genetic variation between replicate populations.

Further analysis revealed highly significant differences in survival rates between generations (Table 3). Parent populations suffered from extinction earlier than F1 and F2 populations (including hybrids and nonhybrids) under environmental deterioration (Fig. 1). This is consistent with parental genomes that constitute productive populations under benign conditions, but that are quickly threatened in changing environments due to lack of genetic variation. Populations of the F2 generation showed significantly superior survival in gradually deteriorating conditions compared with the parental and F1 generation. Lastly, strain identity of the parents also explained some of the variance in offspring survival. The parents used only once had weaker effects than the ‘reference’ parents used in both the intra- and interspecific crosses (Table 1), because the reference parent provided the larger share of genetic raw material (Tables 2 and 3).

Interestingly, F1 populations showed higher survival rates in the first half of the experiment than F2 populations (Fig. 1). This may be an effect of heterosis, that is dominance and overdominance of beneficial alleles that are entirely heterozygous in the F1 generation. At the same time, recessive Dobzhansky–Muller incompatibilities between diverged genomes that are masked in the F1s, may be exposed when they become homozygous in the F2 hybrids, reducing mean fitness (by hybrid breakdown) before being purged by selection.

## Discussion

Hybrid F2 populations persisted longer in deteriorating environments than nonhybrid F2 populations and F2 populations (including both hybrids and nonhybrids) survived more deleterious conditions than both parental and F1 populations. These results confirm that larger amounts of standing genetic variation increase the likelihood of evolutionary rescue (Barrett and Schluter 2008; Agashe et al. 2011; Baskett and Gomulkiewicz 2011). In addition, these data suggest that populations facing rapid environmental change may benefit from introgression and hybridization, even between distant species (the most distant parental strains in our experiment had 14% sequence divergence). This is in agreement with a recent simulation study predicting that introgressive hybridization may be a suitable mechanism for species rescue when certain conditions of assortative mating, hybrid fitness and demographic stochasticity are met (Baskett and Gomulkiewicz 2011).

Evolutionary rescue depends on many environmental and population-specific parameters (Gonzalez and Bell 2013; Carlton et al. 2014), and not every animal and plant taxon has the same predisposition for successful hybridization (Elliot and Crespi 2006). Generally, the relationship between parental genetic distance and hybrid fitness is predicted to be dome-shaped (Price and Waser 1979; Neff 2004). At small crossing distances, for example involving closely related individuals, inbreeding depression can unmask deleterious alleles lowering offspring fitness (Charlesworth and Willis 2009). At large distances, for example involving individuals from divergent populations or different species, outbreeding depression can decrease fitness due to negative epistatic interactions (Dobzhansky 1937; Müller 1942; Coyne and Orr 2004) and the disruption of beneficial gene complexes (Lynch 1991; Edmands 2002). Hybrid fitness is therefore expected to peak at intermediate crossing distances but finding this ‘optimal outbreeding distance’ has proved difficult (Edmands 2007; Robinson et al. 2009; Stelkens et al. 2014b).

Our experiment captured the entire segregational variance generated through hybridization. We tested whether populations, regardless of their mean fitness, contained genotypes showing high fitness in environments inaccessible to the parents. Generally, we expected hybrid populations to have higher variance in fitness but lower mean fitness than nonhybrid populations due to hybrid incompatibilities: for instance, 99% of hybrid genotypes produced from crosses between *S. paradoxus* and *S. cerevisiae* are completely inviable even under benign conditions (Hunter et al. 1996). The ability of yeast to reproduce asexually, allowing high fitness individuals to rapidly produce large populations, represents an obvious difference to most obligate-sexual organisms. One viable, stress-tolerant F2 genotype would have been enough to save a population from extinction in our experiment. Under the same rate of change, smaller and more slowly reproducing sexual populations would suffer from larger environmental stochasticity, and demographic factors may lead to extinction even when populations have the necessary genetic variation to evolve (Lynch and Lande 1993; Hoffmann and Sgro 2011).

The genetic mechanisms enabling hybrids to survive more severe environmental conditions than their parents potentially include transgressive segregation (the extreme over- or underexpression of phenotypic traits due to epistasis and/or the complementation of alleles fixed for opposite signs in the parents; Rieseberg et al. 1999; Stelkens and Seehausen 2009), dominance, overdominance and dosage effects from ploidy-level changes during F1 hybrid meiosis (Selmecki et al. 2009; Pavelka et al. 2010). To understand why some hybrid genotypes survive in high salt environments, we are currently developing a modified RAD-tag sequencing method to elucidate the trait architecture of stress tolerance and to determine the karyotypes of F2 hybrids.

Our results stand in contrast to the many reports of negative effects of recombining divergent genomes, that is hybrid incompatibility (Coyne and Orr 2004; Matute et al. 2010; Moyle and Nakazato 2010; Stelkens et al. 2010; Giraud and Gourbiere 2012). Indeed, hybridization is usually seen as a conservation risk both because it reduces fitness (e.g. Muhlfeld et al. 2009) and because it often leads to a net loss of diversity (Seehausen et al. 2008; Vonlanthen et al. 2012). However, there are also good examples from Darwin's finches (Grant and Grant 2008) and *Helianthus* sunflowers (Rieseberg et al. 2003), showing that hybridization can help adaptation to new niches, as well as evidence that species can expand their climatic ranges as a consequence of introgression from other species (Krehenwinkel and Tautz 2013). Hybridization can even have major macroevolutionary consequences and lead to increased speciation rates in adaptive radiations (Seehausen 2004). One may thus call it a fortunate synergy that when species distributions shift to escape climate change, the opportunities for hybridization resulting in adaptive escape also increase (Hoffmann and Sgro 2011). This may be especially relevant when a native species lacks adaptive potential because its population has already diminished in size and become inbred. As predicted by Baskett and Gomulkiewicz (2011) and in agreement with our results, hybridization in response to environmental change becomes a desirable outcome if it rescues a native population from extinction.

Not many studies have investigated the effect of interspecific hybridization on endangered animal and plant populations in the wild, for the obvious reason that predicting the outcome of a genetic rescue attempt involving hybridization is exceedingly complex. Using heterospecific mates, even from closely related sister species, to boost the genetic variation of a population, might blur the line of what constitutes a native population or species, and constitutes a strongly invasive management strategy that comes with economic, social and political implications (see for instance attempts to rescue the charismatic Florida panther using mates from a different subspecies; Pimm et al. 2006; Hostetler et al. 2013). There are, however, more and more reports on populations at risk that have been intentionally cross-bred with individuals from larger, genetically divergent populations [e.g. in snakes (Madsen et al. 1999), birds (Westemeier et al. 1998), mammals (Pimm et al. 2006) and plants (Willi et al. 2007)]. These studies all encourage genetic approaches to conservation and support the importance of preserving the genetic variability of a species rather than trying to conserve distinct, but often highly inbred local populations or subspecies.

The unprecedented rate of man-made environmental change brings an urgent need to understand how unorthodox mating affects extinction risk. Our data show that the genetic enrichment resulting from hybridization can overcome immediate shortages of genetic variation and help populations to adapt to environmental deterioration, increasing their chances for long-term survival. Whether hybridization benefits an endangered population depends on complex genetic and nongenetic factors (e.g. the degree of epistasis, demography and environmental aspects). An important task for ‘evolutionarily informed’ conservation will therefore be to evaluate whether the positive effects of genetic influx from heterospecific matings outweigh the detrimental effects of outbreeding depression in hybrid populations.

## Data archiving

Raw data for this study will be available from the Dryad Digital Repository: http://doi.org/10.5061/dryad.1jr25.

## Supporting Information

**Figure S1.** Yeast life cycle with non-hybrid and hybrid crossing scheme. **Figure S2.** Serial transfer scheme into deteriorating environments
