# Supplementary material for: Hybridization facilitates evolutionary rescue
Source: Evol Appl. 2014 Sep 25;7(10):1209–17. doi: 10.1111/eva.12214 (PMC4275092; doi:10.1111/eva.12214)
Supplement: Supplementary file 1 [file eva0007-1209-sd1.docx]

**Supplementary Materials**

**Figure S1: Yeast life cycle with non-hybrid and hybrid crossing scheme**

One ‘n’ indicates haploidy, two ‘n’ indicate diploidy, ‘a’ and ‘α‘ indicate different mating types. Asterisks indicate the cell types used in the experiment. (1) Parental haploids divide by mitotic budding from a single cell (2) Haploids from the same clone switch mating type and fusion between *MAT*a haploid and *MAT*α haploid cells occurs. (3) Formation of homozygous parental diploid cells that can divide by mitotic budding. (4) Formation of heterozygous F1 diploid cells that divide by mitotic budding. (5) Under starvation, F1 diploids undergo meiosis, forming haploid spores that germinate and become metabolically active haploid F1 cells. (6) Haploids produced by F1 diploids fuse with others of opposite mating type (7) Formation of F2 diploid cells which can divide by mitotic budding.

**Figure S2: Serial transfer scheme into deteriorating environments**

Shaded areas on 96-well culture plates indicate which wells were inoculated with yeast. Numbers above plates show the amount of NaCl used in growth medium (g/L). Arrows indicate the order of transfers into plates with increasing (or equal) salt concentrations. Numbers of days since the beginning of the experiment are indicated.
